# American Association of Obstetricians and Gynecologists

**Published:** 1907-10

**Authors:** 


**A Monthly Review of Medicine and Surgery.**

*EDITOR:*

WILLIAM WARREN POTTER, M. D.

**All communications, whether of a literary or business nature, books for review and exchanges, should be addressed to the editor 238 Delaware Ave., Buffalo, N. Y.**

Vol. LXIII. OCTOBER, 1907. No. 3

### American Association of Obstetricians and Gynecologists.

WHEN an association of medical men, organised for the scientific study of special topics, reaches its twentieth year its work becomes a fit subject for comment, if not critiism. The association in question held its initial meeting at Buffalo, April 19, 1888, and its first annual meeting at Washington, September 18, 19, and 20, the same year. Fifteen men were present at the initial meeting, who were supported by the coöperation of fourteen others, and by the time of the first annual meeting these had increased to a total of forty, who became foundation members.

Of the forty Founders but thirteen remain on the rolls as Ordinary Fellows, thirteen having resigned, ten are dead, two have been transferred to the Honorary list and two were dropped. The association was denied entrance to the Congress of American Physicians and Surgeons for reasons recited by President Wright in his address published in the fourth volume of the transactions (1891), and which need not be repeated here. Nothing daunted, the association put its hand to the work with redoubled vigor and has made itself one of the most stalwart, progressive, scientific and interesting special medical societies of the land.

Its twentieth annual meeting was held at Detroit, September 17-19, 1907, under the presidency of Professor Robert Tuttle Morris, of New York, and it is no exaggeration to state that it was one of the most interesting, instructive and useful meetings the association has ever held. The papers dealt with live questions, the debates were crisp and aggressive and the entire atmosphere was full of progressive thought. The guiding hand of Dr. Morris was noted from the beginning to the end, and indicated that the association had been in the hands of a master for a year, and now had met bringing the harvest it had garnered during that period, to have the wheat and chaff separated, the threshing process being carried out by brilliant minds in discussions that will redound to the credit of the participants.

It is not our purpose to enter into an analysis of the contributions as these all will be published in the American Journal of Obstetrics for November; but we wish to mention two features of unusual interest. One was the address of Dr. F. Park Lewis, of Buffalo, entitled "Ophthalmia Neonatorum; a Pathologic Anachronism," which was delivered Tuesday evening. Dr. Lewis is chairman of the committee on ophthalmia neonatorum of the American Medical Association and presented this subject by invitation. It has been demonstrated that more than 10% of the blindness in the United States comes from this cause and is absolutely preventable. Furthermore, that by the proper application of prophylactic measures, and particularly by the use of the silver solution according to the technic of Crédé, the blindness resultant from ophthalmia neonatorum can be reduced to a very small fraction of 1%. Hence, it is important for obstetricians, ophthalmologists, public health officers,—indeed for all physicians to reunite in an effort to carry out a prophylaxis that shall wipe out the malady. Puerperal sepsis and ophthalmia neonatorum should belong to a past record. The association took special action on the subject of Dr. Lewis's address.

The second feature for special comment was the annual address of the president. This was delivered extemporaneously from well arranged notes and dealt with the newer questions of surgical technic, which are likely to be recognised in the near future as the only way to the better results that a minimum mortality always hails. Dr. Morris received the plaudits of his audience and his address, at his personal request, was discussed by a number of the members. Taken altogether, it was one of the most satisfactory and entertaining evening meetings ever held by the association.

The banquet given at the Hotel Cadillac, on Wednesday evening, also deserves special mention because of its excellent quality. The table in the form of a hollow square and its central decoration of palms, with its red candelabras, ornamented with large bows of ribbon to correspond, made an effective and artistic setting, while the classic music, rendered by the hotel orchestra, charmed the ear with its artistic and excellent quality. The speeches, too, were crisp, while the ensemble of the banquet was attractive and interest in it was never lost for a moment. The attendance on the third day was unusually large and the closing ceremonies at 3.30 afforded a fitting finale to a brilliant meeting.

The association enjoyed a rare treat during Wednesday's noon intermission. A visit was paid to the great establishment of Messrs. Parke, Davis & Company on invitation of the president, Frank D. Ryan, Esq. Guided by Dr. Larned, of the house, the members, accompanied by the ladies and other guests present, entered a special car and were rapidly driven to the entrance gateway of the plant. A collation first was served, after which Dr. J. H. Carstens made appropriate remarks expressive of the appreciation of the association for the courtesies shown by Mr. Ryan and his assistants. Dr. Larned replied in well chosen words, and then escorted the visitors through the biological laboratories where research work in all its various forms is being conducted.

After an hour and a half spent in the inspection of this marvelous plant the special car was reëntered and the association returned to its duties. It is impossible in this place to give adequate description of the stupendous work that is carrying on in this great institution of biologic research, and it is equally impossible to describe the generous manner in which the association was mentally and physically entertained. The only regret is, that so short a time could be given over to this visit owing to the elaborate scientific program, to the disposal of which the association had committed itself.

The twenty-first annual meeting will be held at Baltimore, Tuesday, Wednesday and Thursday, September 22, 23, and 24, 1908, under the presidency of Dr. E. Gustav Zinke, of Cincinati. The newly elected president is a man of broad culture and a student of the most important and difficult problems connected with his specialty. He has contributed some of the best literature which the science of obstetrics, gynecology, and abdominal surgery contains, and it is probable that the Baltimore meeting of the association under his administration will be of unusual interest.

The other officers elected were: first vice-president, John W. Keefe, Providence; second vice-president, William Alfred Belt Sellman, Baltimore; secretary, William Warren Potter, Buffalo; treasurer, Xavier Oswald Werder, Pittsburg; executive councellors, William Henry Humiston, Cleveland, and Robert Tuttle Morris, New York.

---

THAT President Roosevelt is a many-sided man of large mental equipment is well known. He was seen lately from a new point of view at the opening of the Sixth International Dermatological Congress held in New York, beginning September 9, 1907.

Dr. James S. White of Boston called the congress to order and Surgeon General P. M. Rixey of the United States Navy delivered an address of welcome on behalf of the federal government and President Roosevelt.

Admiral Rixey said that he had been requested by the President to greet the members in his name, to specially welcome the foreign delegates and to wish them a profitable meeting. He said the President was deeply interested in all that concerned the medical profession and embraced every opportunity to secure its advancement. During President Roosevelt's administration he had done a great deal to secure improvements of the medical departments of the government and the government hospitals, and the government now was engaged more than ever in medical research. Dr. Rixey further said:

"It may never be known how much President Roosevelt is interested in and has done for the medical profession until one has been as intimately connected with him as I have. I take this opportunity of saying that in the history of presidents, I may say, rulers, there has been none who has been more interested in its progress than President Roosevelt. I have the deepest sense of our obligations and personal love for the representative of a great people, who stands for justice to all, especially to those physically afflicted."

---

Dr. Ida C. Bender, supervisor of primary grades in the Buffalo school department, recently returned from her visit to London, whither she went to attend the Second International Congress of School Hygiene, bringing back with her many helpful suggestions from which Buffalo schools ought to profit. There were eleven sections, of the congress meeting simultaneously, and she spent most of her time at the conferences that had a direct bearing upon school hygiene, the training of teachers, the care of children, and other subjects of special importance in .connection with her work of supervision in the schools.

"I found," said Dr. Bender in a recent interview, "that Buffalo, or rather America, is ahead in the matter of proper hygienic appliances, ventilation and similar details; but this city especially is far behind other places in the matter of medical inspection, the care of defective children and modern methods along these lines. Here Germany, Sweden, Switzerland and France appear to lead the world.

"One of the very interesting addresses I heard was by Mrs. Humphry Ward, on Vacation Schools, and I also visited her vacation school, where fully 1,000 children are cared for daily, 500 in the morning and an equal number in the afternoon, with a fresh corps of teachers for each session. Most of the work is done out of doors."

Dr. Bender herself spoke at one of the conferences, taking part in the discussions, and introduced a resolution before one of the sections which subsequently was adopted.

The congress was opened by the King's representative, the Right Honorable the Earl of Crewe, lord president of the council, and Dr. Bender said that the inaugural address by the president, Sir Lauder Brunton, was an excellent exposition of the business before the congress.

She was especially interested in the work of the section devoted to medical and hygienic inspections in school, and spoke particularly of an admirable address on medical supervision of infant schools, made by Dr. Marion Hunter of London.

The section devoted to physical education and training in personal hygiene was one that enlisted Dr. Bender's sympathies, and she said that the address made before this section by Dr. Mary Scharlieb, of London, was one of the wisest, most practical and most helpful talks of its kind that she had ever heard. The subject was Physical Development of Adolescent Girls.

Dr. Bender is one of the most accomplished women educators in Buffalo, and we have quoted what she said in a recent newspaper interview for the purpose of inviting attention once more to the importance of medical inspection of schools. We hope that appropriate action will not much longer be delayed by the municipal government relating to this all important topic. It is unfortunate that Buffalo stands so low down on the list of large cities in regard to so vital a question, one that may affect the health and possibly the future lives of so many children.

---

ANNOUNCEMENT through the associated press of the discovery of an antitoxin that will kill diphtheria germs in the living human organism within three minutes has been made at the Ohio State University by Professor Blylle, physiological chemist, as the result of an exhaustive series of tests. The discovery is accredited to Theodore Wolfram, a German chemist, now living here.

---

